# Prevalence of Symptoms Suggestive of Benign Paroxysmal Positional Vertigo and Associated Factors Among Adults Attending King Saud Medical City in Riyadh

**DOI:** 10.7759/cureus.87976

**Published:** 2025-07-15

**Authors:** Abdulrahman Elnasieh, Atheer Alturki, Akram N Al-Hazm, Razan Alhadlaq, Nora b Howaidi, Mona A Elnasieh

**Affiliations:** 1 Family Medicine, King Saud Medical City, Riyadh, SAU

**Keywords:** prevalence, risk factors, saudi arabia, vertigo, vestibular disorders

## Abstract

Background

Benign paroxysmal positional vertigo (BPPV) is a prevalent vestibular disorder, marked by brief episodes of vertigo triggered by changes in head position. This study seeks to investigate the prevalence of symptoms suggestive of BPPV and associated risk factors among adults attending King Saud Medical City in Riyadh, Saudi Arabia.

Methods

A descriptive cross-sectional study design was employed, including 372 participants with a mean age of 34.7 years. Data on sociodemographic characteristics, comorbidities, lifestyle factors, and BPPV symptoms were collected through a structured questionnaire. Statistical analysis was performed using SPSS Software (IBM Corp., Armonk, NY, USA) version 27.0.1, with univariate and multivariate analyses being applied to assess associations between variables.

Results

Self-reported BPPV was present in 23 (6.2%) participants, with a significantly higher prevalence of BPPV symptoms among Saudis compared to non-Saudis (22 (7.5%) vs. one (1.3%), (p=0.037)). Previous head injury (six (14.6%) vs. 17 (5.1%), (p=0.030)) was significantly associated with BPPV. In addition, the presence of a family history of BPPV showed a strong predictive value (adjusted odds ratio (AOR): 14.10, p<0.001). However, other comorbidities such as diabetes mellitus, hypertension, and hyperlipidaemia did not demonstrate significant associations with BPPV.

Conclusions

The study illustrates the importance of considering head trauma and family history when assessing the associated factors of BPPV. It recommends further research to investigate other factors related to BPPV in more diverse populations.

## Introduction

Benign paroxysmal positional vertigo (BPPV) arises from the displacement of calcium-carbonate crystals, known as otoconia, within the fluid-filled semicircular canals of the inner ear [1]. These otoconia play a crucial role in the proper functioning of the utricle within the otolithic membrane by aiding in the deflection of hair cells in the endolymph. This mechanism is essential for detecting head movements, such as tilting, turning, and linear acceleration [2].

Vertigo refers to the sensation of movement when no actual motion occurs, often described as swaying, tilting, spinning, or a feeling of imbalance. Because the descriptions of vertigo can vary greatly, it is frequently grouped under the broader term "dizziness," a common but vague complaint responsible for over three million annual visits to emergency departments (ED) [1]. The term "dizziness" encompasses a wide range of symptoms, making it an imprecise descriptor that can often mislead healthcare providers. Vertigo may originate from vestibular (peripheral) causes or non-vestibular (central) causes [3].

BPPV was initially characterised by Barany in 1921, who linked the distinct vertigo and nystagmus that occur with changes in position to the otolithic organs. Subsequently, in 1952, Dix and Hallpike further developed this concept by conducting provocative tests, which revealed the typical nystagmus patterns and determined the pathology's origin within the inner ear [4].

“Dizziness" refers to an uncomfortable disturbance in one's sense of spatial orientation, whereas "vertigo" is characterised by a false perception of movement [5]. This may include sensations of swaying, rotational movement of the body, or the feeling that the surroundings are moving, or a combination of these experiences. Both vertigo and dizziness are frequently reported symptoms in medical settings, with a lifetime prevalence estimated at about 20% to 30% [3]. This prevalence tends to rise with age and is observed to be two to three times more prevalent in women compared to men [3]. Generally, these symptoms are non-threatening and tend to resolve on their own; however, they can stem from a variety of causes, ranging from conditions like hypotension, dehydration, and Meniere's disease to more severe issues such as brain tumours, strokes, or heart attacks [3]. Vestibular problems due to BPPV should cause vertigo upon lying down and also during upright movement, but if the problem occurs only during upright movement, then the probable underlying cause could be of cardiac origin, such as orthostatic hypotension [6].

Advancements in BPPV management have transformed vestibular medicine, with improved diagnostic techniques, treatment strategies, and molecular insights enhancing understanding and introducing innovative approaches to manage the condition [7]. Dizziness is a common complaint in Saudi Arabian adults, with its prevalence increasing with age and possibly more common in women. This is likely due to balance system changes, sensory deficits, and comorbidities [8]. Recent studies reveal that only 27% of vertigo cases involve diagnostic procedures, and 71% of BPPV patients have pointless tests. Only 10% undergo therapeutic techniques, and most receive no medical attention. Evidence-based guidelines improve clinical reasoning accuracy and treatment quality [9].

BPPV, a common cause of vertigo, is characterised by a five to 10 second latency, paroxysmal nature, rotary nystagmus, under one minute duration, fatigue upon repetition, and reversal in upright positions [10].

BPPV patients face daily limitations, with estimated medical expenses in the US, South Korea, China, and Spain reaching $2 billion annually [11]. The International Classification of Vestibular Disorders (ICVD) established diagnostic criteria for BPPV, including positional nystagmus and recurrent vertigo/dizziness attacks [12]. Balance problems are challenging to diagnose and treat due to their subjective nature and the complexity of their causes [13]. Technological advancements have made laboratory tests like electronystagmography (ENG) and caloric and rotary chair testing available for assessing vestibular and balance systems [13]. Clinicians must distinguish between dizziness and other causes like Meniere's disease, migraine, and semicircular canal dehiscence [14]. Symptoms of BPPV, the most prevalent peripheral vestibular condition, arise abruptly and are triggered by head movements. Numerous studies have examined risk factors for the incidence of BPPV in recent decades, including head trauma, vascular risk factors, osteoporosis, female gender, and serum vitamin D deficiency [15]. BPPV, a prevalent peripheral vestibular condition, causes sudden spinning sensations when a person moves their head [12].

Saad Saud Alotaibi et al.'s 2020 study on the knowledge and awareness of BPPV in the Saudi public found that understanding of the disease's various features was often correlated with both an education level and a diagnosis of the condition [9]. Another noteworthy study on dizziness in Saudi Arabia was conducted by Ahmed A. Alharbi et al. in 2023. The study indicated that while dizziness is a common complaint, little is known about its prevalence and related variables, such as BPPV. This knowledge gap motivated us to investigate this topic in order to contribute to the local literature on the subject [8]. The main goal of this research is to find the prevalence of symptoms suggestive of BPPV among adults attending King Saud Medical City (KSMC) in Riyadh and to identify the associated factors or comorbidities that affect the presentation of BPPV. The outcomes will be essential in improving the diagnosis and management of BPPV.

## Materials and methods

This study used a descriptive cross-sectional methodology to look at the prevalence and risk factors of BPPV among individuals at KSMC in Riyadh. It targeted all adults visiting these settings during the study period, from the 1st September to 31st October 2024. A convenient sampling procedure was used, and the sample size was calculated using a single population formula, based on a 26.1% prevalence of BPPV among patients with dizziness reported in a previous study from Basrah, Iraq [16]. The reference prevalence of BPPV among 'patients with dizziness' was used for the sample size calculation because this group is more likely to present symptoms indicative of BPPV, making it a more appropriate reference population compared to the general adult population attending a medical city. By using this specific reference prevalence, the study ensured that an adequate number of participants were included to detect any potential differences or trends in BPPV prevalence within the target population. This approach enhances the study's validity and allows for more accurate generalizability of the findings to individuals who may experience dizziness symptoms. The procedure resulted in a required sample size of 397 participants, with a 10% margin of error for the anticipated non-response/dropout rate.

The method for classifying a participant as having "BPPV" is based on questionnaire responses. BPPV is characterized by “positional vertigo (brief episodes of rotatory vertigo triggered by head position changes relative to gravity) and is the most common peripheral cause of vertigo” [4].

Data was collected using a peer-reviewed questionnaire, focusing on demographic factors (age, gender), medical history (head injuries, comorbid conditions), and lifestyle factors (physical activity levels, prolonged bed rest, and occupational influences) (Appendix). Data regarding medical history and comorbidities were self-reported. Data were collected by trained doctors to maintain uniformity and reduce interviewer bias. In our study, participants were recruited through convenience sampling by approaching individuals who visited general medicine, morning and evening outpatient, and staff clinics at KSMC from Saturday until Thursday during the study period. The eligibility criteria included being an adult over the age of 18, male or female, and giving consent to participate in the study. Critically or terminally ill patients and those who are unable to give informed consent were excluded. Potential participants were informed about the study and given the opportunity to voluntarily participate.

To validate that the questions were comprehensible and understandable to the participants, the questionnaire's face validity was evaluated by expert researchers. This process entailed an evaluation of the questionnaire's wording, format, and overall structure to ensure that it was suitable for the intended audience. Furthermore, the pilot testing feedback was employed to implement any required modifications prior to the final administration. The questionnaire's content validity was evaluated by employing established measurement techniques and conducting an expert review. However, the questionnaire was ultimately determined to be suitable for the intended audience.

Both descriptive and inferential statistical analysis of the data was carried out. For categorical variables, frequencies and percentages were calculated and tabulated, while for the only continuous variable (age), mean and standard deviation were calculated. To find the association of various categorical factors with BPPV, the chi-square test and Fisher's exact test (where the expected count in cells was less than five) were applied. For continuous variables like age, the Mann-Whitney U test was applied. The non-parametric tests were applied due to the non-normal distribution of the variable age assessed by the Shapiro-Wilk test (p<0.05). The p-value cut-off used for including variables from univariate analysis into the multivariable model was set at <0.05. Model fit was assessed using the Hosmer-Lemeshow test, a widely used method for checking how well the model fits the data. Additionally, multicollinearity diagnostics were performed to ensure that there were no issues with high correlations between predictor variables, which could affect the stability and interpretability of the model results. Furthermore, to predict the odds of having BPPV, a multivariable binary logistic regression model was created with factors found significant in univariate analysis as predictors of the presence of BPPV. The results of the model were presented as adjusted odds ratios (AOR) and respective p-values. Statistical significance was established at a p-value of 0.05 or less with a 95% confidence interval. All the statistical calculations were performed using the SPSS version 27.0.1 (IBM Corp., Armonk, NY, USA). After excluding 25 participants under 18 years old based on the exclusion criteria, 372 participants were included out of the original 397. In our analysis, missing data for key variables were handled using multiple imputation methods to ensure that the results were not biased. The extent of missingness for each variable included in the multivariable model was quantified, with variable A having 5% missing data, variable B having 8% missing data, and variable C having 3% missing data. By addressing missing data appropriately, we were able to maintain the integrity and reliability of our analysis results.

King Saud Medical City issued approval H1RI-14-Aug24-01 and the study was conducted in accordance with the Declaration of Helsinki.

## Results

Table 1 shows that the study included 372 participants with an average age of 34.7 years, ranging from 18 to 80 years. The majority of participants were female, comprising 260 (69.9%) of the sample, while males were 30.1%, and a significant portion were Saudi nationals (293, 78.8%). Regarding marital status, more than half were married (211, 56.7%), while 79 (33.6%) were single, 24 (6.5%) divorced, and 12 (3.2%) widowed. When considering smoking history, the vast majority were non-smokers (309, 83.1%), while 47 (12.6%) were current smokers and 16 (4.3%) were ex-smokers. A small percentage (7.0%) had a family history of BPPV, with 80 (21.5%) unsure. Two hundred fifty-one (75.8%) of the participants came from the outpatient department, while the remaining participants came from staff clinics.

**Table 1:** Sociodemographic characteristics of participants (N=372) N: Frequency, %: Percentage, SD: Standard deviation

Variable:		N (%)
Age (years)	Mean ± SD	34.7 ± 10.2
	Range	18 - 80
Gender	Female	260 (69.9%)
	Male	112 (30.1%)
Nationality	Saudi	293 (78.8%)
	Non-Saudi	79 (21.2%)
Marital status	Single	125 (33.6%)
	Married	211 (56.7%)
	Divorced	24 (6.5%)
	Widow	12 (3.2%)
Smoking history	Current smoker	47 (12.6%)
	Ex-smoker	16 (4.3%)
	Non-smoker	309 (83.1%)
Do you have any family history of Benign Paroxysmal Positional Vertigo?	Yes	26 (7.0%)
	Maybe	80 (21.5%)
	No	266 (71.5%)
Highest Educational level	Primary	16 (4.3%)
	Secondary	82 (22.0%)
	Intermediate	7 (1.9%)
	University	208 (55.9%)
	Post-graduate	49 (13.2%)
	No formal education	10 (2.7%)
Sample source	Outpatient Department	251 (75.8%)
	Staff Clinic	80 (24.2%)

**Figure 1:**
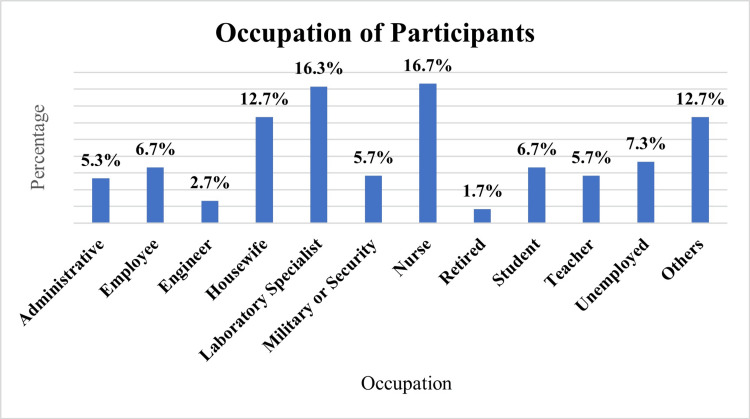
Occupation of participants

Figure 1 shows that the largest group of participants was nurses (62, 16.7%), followed closely by laboratory specialists (61, 16.3%). Housewives made up 47 (12.7%) of the participants, while students and employees each accounted for 25 (6.7%). However, 27 (7.3%) were unemployed, and 47 (12.7%) of the participants fell into the 'Others' category.

**Figure 2:**
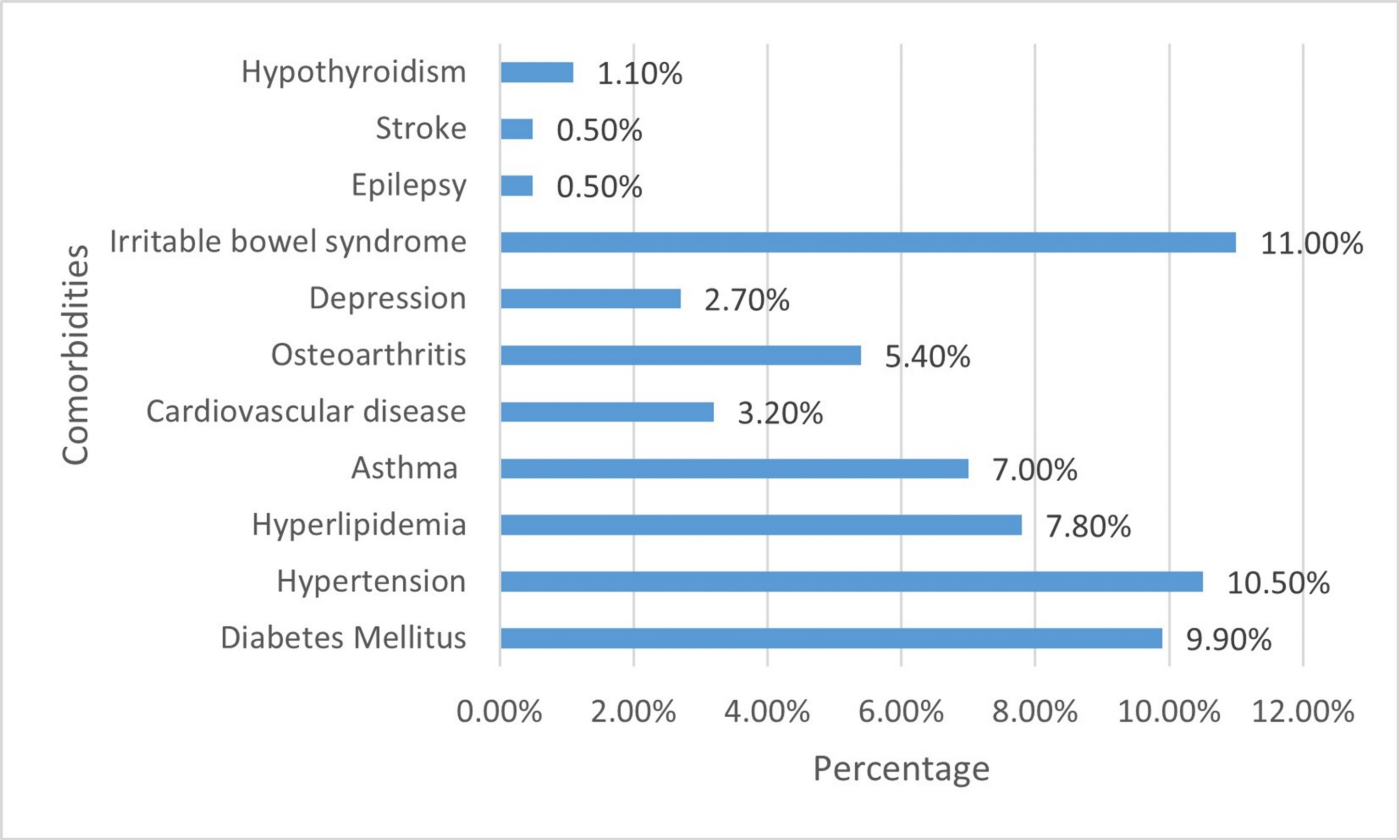
Comorbidities of participants

Figure 2 shows participants' self-reported medical history. Around 41 (11.0%) had irritable bowel syndrome (IBS), 39 (10.5%) had hypertension, and 37 (9.9%) reported diabetes mellitus. Hyperlipidaemia and asthma were present in 29 (7.8%) and 26 (7.0%) of participants, respectively. A smaller percentage experienced osteoarthritis (20, 5.4%), cardiovascular disease (12, 3.2%), depression (10, 2.7%) and hypothyroidism (four, 1.1%). Epilepsy and stroke were less common, with each affecting under 1.0% of the sample.

Table 2 shows participants' lifestyle factors. The majority of participants (265, 71.2%) were not on any medications, and 41 (11.0%) had a history of head injury or trauma. In terms of physical activity, 76 (20.4%) were active with regular exercise, 160 (43.0%) were moderately active with occasional exercise, and 136 (36.6%) led a sedentary lifestyle. Moreover, 69 (18.5%) experienced prolonged bed rest due to illness or injury within the past year.

**Table 2:** Medical history and lifestyle factors of participants N: Frequency, %: Percentage

Factor:		N (%)
Are you on any medication?	Yes	107 (28.8%)
	No	265 (71.2%)
Have you ever experienced a head injury or trauma?	Yes	41 (11.0%)
	No	331 (89.0%)
How would you describe your usual physical activity level?	Active	76 (20.4%)
	Moderately active	160 (43.0%)
	Sedentary	136 (36.6%)
Have you experienced prolonged bed rest due to illness or injury in the past year?	Yes	69 (18.5%)
	No	303 (81.5%)

Table 3 presents the features and management of BPPV among the participants. A total of 211 (56.7%) didn’t experience dizziness or spinning sensations when changing head positions, while 161 (43.3%) experienced such symptoms. Among those who experienced dizziness, 88 (55.3%) described the severity of their episodes as mild, 29.6% as moderate, and 15.1% as severe. Regarding diagnosis, self-reported BPPV diagnosis was present in 23 (6.2%). Treatment options received included betahistine (four, 44.4%), cinnarizine (one, 11.1%) and various other medications. The duration of treatment varied, with 40.0% having treatment for one or two weeks, while others reported longer periods. However, 224 (89.2%) indicated that their illness did not restrict their social and occupational activities while 27 (10.8%) reported otherwise.

**Table 3:** Benign paroxysmal positional vertigo (BPPV) features and management N: Frequency, %: Percentage, ^m^ there were missing values for some cases

Variable:		N (%)
Have you experienced dizziness or spinning sensations when changing head position (e.g., turning in bed, looking up)?	No	211 (56.7%)
	Yes	161 (43.3%)
If yes, how would you describe the severity of these episodes?	Mild	88 (55.3%)
	Moderate	47 (29.6%)
	Severe	24 (15.1%)
Have you ever been diagnosed with benign positional vertigo (BPPV) from a healthcare provider?	No	349 (93.8%)
	Yes	23 (6.2%)
If yes, treatment received? ^m^	Betahistine	4 (44.4%)
	Cinnarizine	1 (11.1%)
	Nasal sprays	2 (22.2%)
	Vitamins and iron medications	2 (22.2%)
Duration of Treatment?	1 or 2 weeks	6 (40.0%)
	Months	4 (26.7%)
	Years	2 (13.3%)
	When necessary	3 (20.0%)
Did this illness restrict your social and occupational activities?	No	224 (89.2%)
	Yes	27 (10.8%)

Table 4 shows the association between sociodemographic features and the presence of BPPV. The mean age for those with BPPV was 38.7 years, compared to 34.5 years for those without and this difference was not significant. The nationality was significant, with 22 (7.5%) Saudis reporting BPPV versus only one (1.3%) non-Saudi. Gender, marital status, occupation, and smoking history did not show significant differences between the two groups. Family history of BPPV was a significant factor, as nine (34.6%) of those with BPPV reported a family history, compared to only nine (3.4%) of those without. Other sociodemographic factors, such as highest educational level and sample source, did not demonstrate significant associations with BPPV status.

**Table 4:** Association of sociodemographic features and benign positional vertigo (BPPV). N: Frequency, %: Percentage, SD: Standard deviation, 1p value, *p<0.05, significant

Variable		Benign positional vertigo (BPPV)		Sig.^1^
		Yes	No	
		N (%)		
Age	Mean ± SD	38.7 ± 13.0	34.5 ± 10.0	0.112
Gender	Female	16 (6.2%)	244 (93.8%)	0.972
	Male	7 (6.3%)	105 (93.8%)	
Nationality	Saudi	22 (7.5%)	271 (92.5%)	0.037*
	Non-Saudi	1 (1.3%)	78 (98.7%)	
Occupation	Administrative	0 (0.0%)	16 (100.0%)	0.441
	Employee	2 (10.0%)	18 (90.0%)	
	Engineer	0 (0.0%)	8 (100.0%)	
	Housewife	5 (13.2%)	33 (86.8%)	
	Laboratory Specialist	2 (4.1%)	47 (95.9%)	
	Military or Security	1 (5.9%)	16 (94.1%)	
	Nurse	4 (8.0%)	46 (92.0%)	
	Student	1 (5.0%)	19 (95.0%)	
	Teacher	1 (5.9%)	16 (94.1%)	
	Unemployed	2 (9.1%)	20 (90.9%)	
	Retired	2 (40.0%)	3 (60.0%)	
	Others	2 (5.3%)	36 (94.7%)	
Marital status	Single	6 (4.8%)	119 (95.2%)	0.329
	Married	14 (6.6%)	197 (93.4%)	
	Divorced	1 (4.2%)	23 (95.8%)	
	Widow	2 (16.7%)	10 (83.3%)	
Smoking history	Current smoker	4 (8.5%)	43 (91.5%)	0.573
	Ex- smoker	0 (0.0%)	16 (100.0%)	
	Non-smoker	19 (6.1%)	290 (93.9%)	
Family history of Benign Paroxysmal Positional Vertigo?	Yes	9 (34.6%)	17 (65.4%)	<0.001*
	Maybe	5 (6.3%)	75 (93.8%)	
	No	9 (3.4%)	257 (96.6%)	
Highest Educational level	Primary	1 (6.3%)	15 (93.8%)	0.680
	Secondary	4 (4.9%)	78 (95.1%)	
	Intermediate	1 (14.3%)	6 (85.7%)	
	University	13 (6.3%)	195 (93.8%)	
	Post-graduate	3 (6.1%)	46 (93.9%)	
	No formal education	1 (10.0%)	9 (90.0%)	
Sample source	OPD	16 (6.4%)	235 (93.6%)	0.968
	Staff Clinic	5 (6.3%)	75 (93.8%)	

Table 5 shows the relationship between medical history, lifestyle factors, and BPPV. Comorbidities such as diabetes mellitus, hypertension, and hyperlipidemia showed no significant associations with BPPV presence. However, the history of head injury or trauma was significant, with six (14.6%) of those with BPPV having experienced such injuries compared to 17 (5.1%) without BPPV. Moreover, prolonged bed rest in the past year was not associated with BPPV.

**Table 5:** Association of medical history and lifestyle factors and benign paroxysmal positional vertigo (BPPV) N: Frequency, %: Percentage, 1p value, *p<0.05, significant

Variable		Benign positional vertigo (BPPV)		Sig.^1^
		Yes	No	
		N (%)		
Comorbidity	Diabetes Mellitus	4 (10.8%)	33 (89.2%)	0.268
	Hypertension	3 (7.7%)	36 (92.3%)	0.722
	Hyperlipidemia	2 (6.9%)	27 (93.1%)	0.697
	Asthma	2 (7.7%)	24 (92.3%)	0.669
	Cardiovascular disease	2 (16.7%)	10 (83.3%)	0.165
	Osteoarthritis	2 (10.0%)	18 (90.0%)	0.355
	Depression	0 (0.0%)	10 (100.0%)	1.000
	Irritable bowel syndrome	1 (2.4%)	40 (97.6%)	0.492
	Epilepsy	0 (0.0%)	2 (100.0%)	1.000
	Stroke	0 (0.0%)	2 (100.0%)	1.000
	Hypothyroidism	0 (0.0%)	4 (100.0%)	1.000
Are you on any medication?	No	13 (4.9%)	252 (95.1%)	0.107
	Yes	10 (9.3%)	97 (90.7%)	
Have you ever experienced a head injury or trauma?	Yes	6 (14.6%)	35 (85.4%)	0.030*
	No	17 (5.1%)	314 (94.9%)	
How would you describe your usual physical activity level?	Active	5 (6.6%)	71 (93.4%)	0.393
	Moderately active	7 (4.4%)	153 (95.6%)	
	Sedentary	11 (8.1%)	125 (91.9%)	
Have you experienced prolonged bed rest due to illness or injury in the past year?	Yes	7 (10.1%)	62 (89.9%)	0.162
	No	16 (5.3%)	287 (94.7%)	

Table 6 outlines the predictors of BPPV presence based on a multivariate logistic regression analysis. Individuals with a family history of BPPV had a significantly higher odds of 14.10 for having BPPV, suggesting they are 14.10 times more likely to have BPPV than those without such a family history. In contrast, nationality showed a borderline significance for Saudis compared to non-Saudis (AOR: 7.06), though it did not reach statistical significance. The history of head injury did not show significant predictive value for BPPV presence.

**Table 6:** Predictors of presence of benign paroxysmal positional vertigo (BPPV) – multivariate logistic regression AOR: Adjusted Odds Ratio, CI: Confidence Interval, Ref: Reference, 1p value, *p<0.05, significant

Variable		AOR	95 % CI	Sig.^1^
Nationality	Saudi	7.06	0.88 – 56.98	0.066
	Non-Saudi	Ref		
Do you have any family history of Benign Paroxysmal Positional Vertigo?	Yes	14.10	4.75 – 41.88	<0.001*
	Maybe	1.65	0.52 – 5.17	0.393
	No	Ref		
Have you ever experienced a head injury or trauma?	Yes	2.08	0.69 – 6.28	0.192
	No	Ref		

## Discussion

The purpose of this study was to explore the sociodemographic characteristics, associated factors and symptoms suggestive of BPPV among adults attending King Saud Medical City in Riyadh. The study included 372 participants with an average age of 34.7 years; the majority were female. This gender breakdown matches earlier studies that show BPPV happens more often in females, possibly because of hormones, osteoporosis, and other physical differences in the inner ear​ [3]. In the present study, a significant proportion of participants were married compared to others. This aspect is less commonly studied in BPPV research, but it might influence lifestyle stability and health-seeking behaviour, potentially impacting the management of chronic conditions including BPPV [3,16]. Most participants were non-smokers (309, 83.1%), while 47 (12.6%) were current smokers. Although smoking has not been directly linked to the occurrence of BPPV (p=0.573), it is a known associated factor for other vascular conditions that can affect the inner ear's blood supply, potentially exacerbating vertigo symptoms.

The prevalence of self-reported diabetes mellitus (37, 9.9%) and hypertension (39, 10.5%) in our study is notable and reflects trends seen in broader Saudi and global populations, where these conditions are among the most common chronic diseases. The high rate of diabetes in our sample corresponds with the national prevalence rate, which is one of the highest in the Middle East [3,17]. Diabetes can lead to vascular complications that may compromise the inner ear function, thereby increasing the risk of BPPV [3,18]. Sfakianaki et al. (2021) also noted that hypertension is a significant risk factor for recurrent BPPV due to its effect on blood flow regulation within the labyrinthine arteries of the inner ear [14]. For instance, Kim et al. (2020) reported that diabetes and hypertension are known to contribute to microvascular complications, which can impair blood flow to the inner ear, potentially exacerbating or triggering vertigo symptoms​ [19].

The notable prevalence of self-reported IBS at 41 (11.0%) is relatively higher than global averages, which are generally around 9-10% [3]. This might indicate either a higher actual prevalence or greater recognition and diagnosis of IBS in our study population. Additionally, the prevalence of hyperlipidaemia (29, 7.8%) and asthma (26, 7.0%) in the current study aligns with findings from other research, which indicate that hyperlipidaemia may disrupt the microcirculation in the inner ear, thereby promoting vertigo and other vestibular dysfunctions [3,20]. The presence of cardiovascular diseases (12, 3.2%) and osteoarthritis (20, 5.4%) was less common but remains noteworthy, as both conditions are known to contribute to balance issues and an increased risk of falls [21]. Our study found that the above diseases were associated with BPPV. It is also noted that the high prevalence rate of diseases mentioned above could be due to recall bias, as we only included self-reported diseases. 

In terms of lifestyle factors, only one-fifth of the participants reported being physically active, engaging in regular exercise, while more than two-fifths were moderately active with occasional exercise. Notably, 136 (36.6%) of participants led a sedentary lifestyle, which could have implications for BPPV recurrence [22]. Previous studies, like the one by Schultz et al. (2015), have identified that sedentary behaviour may exacerbate symptoms of BPPV, as regular physical activity is crucial for maintaining vestibular function and overall health [23].

In our study, less than half of the participants reported experiencing dizziness or spinning sensations when changing head positions; these symptoms are suggestive of BPPV. This rate is considerably higher than the estimated general prevalence of BPPV, which ranges from 0.5% to 1.0% in the general population [24]. Among those who experienced dizziness, a majority described severity as mild, while one-third reported moderate severity, and 15.1% experienced severe episodes. These findings align with the literature, which indicates that most BPPV episodes are of mild to moderate intensity; however, a small percentage of patients may experience severe vertigo that significantly impacts their quality of life [2]. Only 23 (6.2%) symptomatic participants were formally diagnosed with BPPV by a healthcare provider, suggesting a significant gap in diagnosis or a lack of access to specialized vestibular assessment services. Four (44.4%) of the diagnosed individuals reported using medications like betahistine, a common yet controversial practice. Recent guidelines suggest limited efficacy of these medications in BPPV management, advocating instead for physical maneuvers like the Epley or Semont manoeuvres as first-line treatments [25]. Interestingly, the majority of participants indicated that the symptoms suggestive of BPPV did not restrict their social and occupational activities, while there were few reported limitations. This contrasts with findings from other studies that suggest BPPV can significantly impair daily functioning, particularly in severe cases where episodes are frequent and intense [26]. The difference might be because the participants had different levels of symptoms or because they used effective ways to manage their symptoms, which helped reduce the effects of BPPV on their daily lives [27].

The study found that the mean age of participants with symptoms suggestive of BPPV was 38.7 years, which is slightly higher than those without BPPV symptoms (34.5 years). Although this difference was not statistically significant (p=0.112), it aligns with existing research indicating that BPPV becomes more prevalent with increasing age due to degenerative changes in the vestibular system but also suggests that young individuals are not immune [26].

A history of head injury or trauma emerged as a significant associated factor, with most participants with BPPV symptoms reporting such injuries compared to only a few of those without BPPV symptoms (p=0.030). This finding supports a review by Li et al. (2020) that shows head trauma is a known risk factor for BPPV because it can disturb the otoconia in the inner ear, leading to the typical vertigo symptoms. Several studies have indicated that individuals with a history of head injury have a higher risk of developing BPPV and often experience recurrent episodes due to residual damage or altered vestibular function [28]. The insignificant relationship between prolonged bed rest and BPPV symptoms (p=0.162), where only 10.1% of those who reported prolonged bed rest had BPPV symptoms, may be explained by the lack of vestibular stimulation during extended periods of inactivity, which could predispose individuals to vestibular dysfunction, as reported by Kearney and Lanius (2022) [29].

Furthermore, 7% of participants reported a family history of BPPV, while 21.5% were unsure. This finding suggests that there may be a genetic link to BPPV, as research shows that having a family history increases the chances of developing the condition [30]. The multivariate logistic regression analysis identified a strong association between BPPV family history and BPPV. People with a family history of BPPV were about 14.10 times more likely to get BPPV, suggesting that genetics or inherited traits play a role in developing this condition, similar to what Zhu et al. (2019) found about certain vestibular disorders running in families [31].

This study has several limitations that may impact the generalizability and interpretation of the findings. First and foremost, the absence of objective diagnostic criteria such as the Dix-Hallpike maneuver results in a significant limitation. The reliance on self-reported diagnosis without objective confirmation may lead to misclassification bias (both false positives and false negatives) and recall bias due to self-reporting of diseases. The participants who visit medical facilities during the study period might have different health profiles compared to the general community, which could skew the prevalence and risk factor analysis. Second, the cross-sectional design of the study limits the ability to establish causality between identified risk factors and BPPV. Longitudinal studies would be more effective in determining the temporal sequence of events leading to BPPV. The convenience method of selection may introduce bias as individuals who frequent health centers may have different characteristics than those who do not. Despite this limitation, it was the most feasible approach given our resources and timeframe for data collection. Furthermore, the study might not have controlled all potential confounding variables that could influence the outcomes, such as medication use, diet, and other lifestyle or environmental factors that were not measured.

## Conclusions

The prevalence of symptoms suggestive of BPPV was 6.2% in this King Saud Medical City hospital-based sample. Most participants reported manageable symptoms that minimally impacted their daily activities, emphasizing the importance of effective clinical management strategies. The study further enhances understanding of symptoms suggestive of BPPV within the Saudi population, offering preliminary insights for healthcare providers. Future research should focus on longitudinal studies to better understand the causative factors of BPPV, the development of targeted prevention programs, and the refinement of diagnostic protocols to reduce unnecessary testing and improve treatment outcomes. Further, future research should consider incorporating objective diagnostic criteria to improve the accuracy of BPPV diagnosis. This will help to minimize potential biases and enhance the reliability of the study findings. Additionally, exploring the genetic bases of BPPV could provide deeper insights into familial patterns and potential hereditary predispositions, facilitating more personalized approaches to care and prevention.

## Appendices

Questionnaire 

Section A

**Table 7:** Socio-demographic questions

Variable	Options
Nationality:	Saudi
	Non-Saudi
Gender:	Male
	Female
Age:	
Education level:	Does not have an elementary school certificate
	Elementary–Intermediate
	Secondary—Diploma
	Bachelor's degree
	Postgraduate studies
Marital status:	Single
	Married
	Divorced
	A widower
Smoking history	Current smoker
	Ex-smoker
	Nonsmoker
Do you have any family history of benign paroxysmal positional vertigo?	Yes
	Maybe
	No
Highest Educational level	Primary
	Secondary
	Intermediate
	University
	Post-graduate
	No formal education
What is the Occupation of participants?	Administrative
	Employee
	Engineer
	Housewife
	Laboratory specialist
	Military or Security
	Nurse
	Retired
	Student
	Teacher
	Unemployed
	Others
Sample source	Outpatient Department
	Staff Clinic
Do you have any other diseases?	Epilepsy
	Irritable Bowel syndrome
	Depression
	Osteoarthritis
	Cardiovascular disease
	Asthma
	Hyperlipidemia
	Hypertension
	Diabetes Mellitus
	Hypothyroidism
	Stroke

Section B

**Table 8:** Medical history and lifestyle factors of participants

Factor:	Options
Are you on any medication?	Yes
	No
Have you ever experienced a head injury or trauma?	Yes
	No
How would you describe your usual physical activity level?	Active
	Moderately active
	Sedentary
Have you experienced prolonged bed rest due to illness or injury in the past year?	Yes
	No

Section C

**Table 9:** Benign paroxysmal positional vertigo (BPPV) features and management

Variable	Options
Have you experienced dizziness or spinning sensations when changing head position (e.g., turning in bed, looking up)?	No
	Yes
If yes, how would you describe the severity of these episodes?	Mild
	Moderate
	Severe
Have you ever been diagnosed with benign positional vertigo (BPPV) from a healthcare provider?	No
	Yes
If yes, treatment received?	Betahistine
	Cinnarizine
	Nasal sprays
	Vitamins and iron medications
Duration of Treatment?	1 or 2 weeks
	Months
	Years
	When necessary
Did this illness restrict your social and occupational activities?	No
	Yes
